# 2-[(*E*)-(2-Amino­phen­yl)imino­meth­yl]-5-(dimethyl­amino)phenol

**DOI:** 10.1107/S1600536809015773

**Published:** 2009-05-07

**Authors:** Yan-Hong Yu, Kun Qian

**Affiliations:** a Jiangxi Key Laboratory of Organic Chemistry, Jiangxi Science and Technology Normal University, Nanchang 330013, People’s Republic of China; b Academic Administration of JiangXi University of Traditional Chinese Medicine, Nanchang 330047, People’s Republic of China

## Abstract

The mol­ecule of the title compound, C_17_H_21_N_3_O, displays a *trans* configuration with respect to the C=N double bond. The dihedral angle between the planes of the two benzene rings is 50.96 (11)° and a strong intra­molecular O—H⋯N hydrogen bond is present. An inter­molecular N—H⋯O hydrogen-bonding inter­action stabilizes the crystal structure.

## Related literature

For general background to the properties of Schiff base compounds, see: Weber *et al.* (2007 ▶); Chen *et al.* (2008 ▶); May *et al.* (2004 ▶). For the structure of a related compound, see: Elmah *et al.* (1999 ▶).
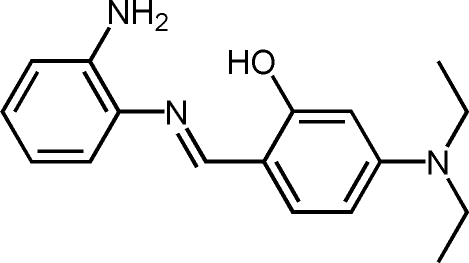


## Experimental

### 

#### Crystal data

C_17_H_21_N_3_O
*M* *_r_* = 283.37
Orthorhombic, 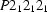

*a* = 6.5904 (13) Å
*b* = 12.703 (3) Å
*c* = 18.538 (4) Å
*V* = 1552.0 (6) Å^3^
*Z* = 4
Mo *K*α radiation
μ = 0.08 mm^−1^
*T* = 293 K
0.20 × 0.20 × 0.20 mm

#### Data collection

Rigaku SCXmini diffractometer
Absorption correction: multi-scan (*CrystalClear*; Rigaku, 2005 ▶) *T*_min_ = 0.973, *T*_max_ = 0.979
16156 measured reflections
2061 independent reflections
1996 reflections with *I* > 2σ(*I*)
*R*_int_ = 0.063

#### Refinement

*R*[*F* ^2^ > 2σ(*F* ^2^)] = 0.058
*wR*(*F* ^2^) = 0.176
*S* = 1.04
2061 reflections
194 parameters
H atoms treated by a mixture of independent and constrained refinement
Δρ_max_ = 0.13 e Å^−3^
Δρ_min_ = −0.14 e Å^−3^

### 

Data collection: *CrystalClear* (Rigaku, 2005 ▶); cell refinement: *CrystalClear*; data reduction: *CrystalClear*; program(s) used to solve structure: *SHELXS97* (Sheldrick, 2008 ▶); program(s) used to refine structure: *SHELXL97* (Sheldrick, 2008 ▶); molecular graphics: *SHELXTL* (Sheldrick,2008 ▶); software used to prepare material for publication: *SHELXL97*.

## Supplementary Material

Crystal structure: contains datablocks I, global. DOI: 10.1107/S1600536809015773/rz2315sup1.cif

Structure factors: contains datablocks I. DOI: 10.1107/S1600536809015773/rz2315Isup2.hkl

Additional supplementary materials:  crystallographic information; 3D view; checkCIF report

## Figures and Tables

**Table 1:** Hydrogen-bond geometry (Å, °)

*D*—H⋯*A*	*D*—H	H⋯*A*	*D*⋯*A*	*D*—H⋯*A*
N3—H3*B*⋯O1^i^	0.86	2.55	3.395 (4)	167
O1—H1*A*⋯N2	0.86 (5)	1.82 (5)	2.638 (4)	157 (4)

Symmetry code: (i) 
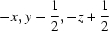
.

**Fig. 1.**
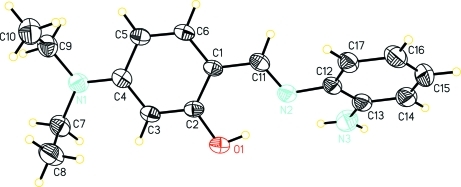
The molecular structure of the title compound with the atomic numbering scheme. Displacement ellipsoids are drawn at the 30% probability level.

**Fig. 2.**
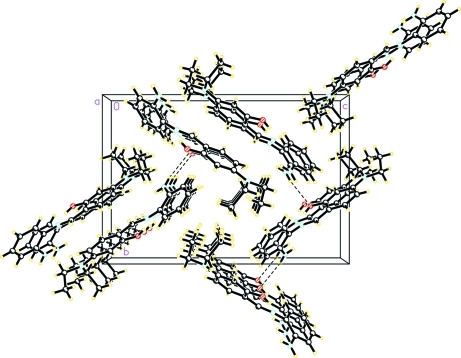
Packing diagram of the title compound, showing the structure along the a axis. Intermolecular N—H···O hydrogen bonds are shown as dashed lines.

## supplementary crystallographic information

### Comment

Schiff base compounds have received considerable attention for many years, primarily due to their importance in the development of coordination chemistry related to magnetism (Weber *et al.*, 2007), catalysis (Chen *et al.*, 2008) and biological processes (May *et al.*,2004). Our group is interested in the synthesis and preparation of Schiff bases. Here, we report the synthesis and crystal structure of the title compound.

The molecular structure of the title compound is shown in Fig. 1. The dihedral angle between the mean planes of the two aromatic rings is 50.96 (11)°, indicating that the Schiff-base ligand adopts a non-planar conformation. As expected, the molecule displays a *trans* configuration about the central C11═N2 bond. Bond lengths and angles observed in the structure are in normal ranges and comparable with those of a related Schiff base compound (Elmah *et al.*, 1999). The hydroxy group is involved as donor in a strong intramolecular O—H···N hydrogen bond (Table 1) and as acceptor in an weak intermolecular N—H···O hydrogen interaction (Fig. 2, Table 1).

### Experimental

Benzene-1,2-diamine (0.59 g, 5 mmol) and 4-(diethylamino)-2-hydroxybenzaldehyde (0.965 g, 5 mmol) were dissolved in methanol (15 ml). The mixture was heated to reflux for 6 h, then cooled to room temperature, then the solution was filtered and dried (yield 84%). Crystals of the title compound suitable for X-ray diffraction analysis were grown by slow evaporation of an ethanol solution. Esi-MS: calcd for C_17_H_21_N_3_O + H *m*/*z* 283.37, found 284.72.

### Refinement

The H atom of the hydroxy group was found in a difference Fourier map and refined freely. The other H atoms were placed geometrically and treated as riding atoms, with N—H =0.86 Å, C—H = 0.93–0.97 Å, and with *U*iso(H) = 1.2 *U*eq(C, N) or 1.5 *U*eq(C) for methyl H atoms. In the absence of significant anomalous scattering effects, 1505 Friedel pairs were merged in the final refinement.

### Crystal data

**Table:**

C_17_H_21_N_3_O	*F*(000) = 608
*M**_r_* = 283.37	*D*_x_ = 1.213 Mg m^−^^3^
Orthorhombic, *P*2_1_2_1_2_1_	Mo *K*α radiation, λ = 0.71073 Å
Hall symbol: P 2ac 2ab	Cell parameters from 1474 reflections
*a* = 6.5904 (13) Å	θ = 3.1–27.8°
*b* = 12.703 (3) Å	µ = 0.08 mm^−^^1^
*c* = 18.538 (4) Å	*T* = 293 K
*V* = 1552.0 (6) Å^3^	Prism, yellow
*Z* = 4	0.20 × 0.20 × 0.20 mm

### Data collection

**Table:**

Rigaku SCXmini diffractometer	3566 independent reflections
Radiation source: fine-focus sealed tube	1996 reflections with *I* > 2σ(*I*)
graphite	*R*_int_ = 0.063
Detector resolution: 13.6612 pixels mm^-1^	θ_max_ = 27.5°, θ_min_ = 3.2°
ω scans	*h* = −8→8
Absorption correction: multi-scan (*CrystalClear*; Rigaku, 2005)	*k* = −16→16
*T*_min_ = 0.973, *T*_max_ = 0.979	*l* = −24→24
16156 measured reflections	

### Refinement

**Table:**

Refinement on *F*^2^	Primary atom site location: structure-invariant direct methods
Least-squares matrix: full	Secondary atom site location: difference Fourier map
*R*[*F*^2^ > 2σ(*F*^2^)] = 0.058	Hydrogen site location: inferred from neighbouring sites
*wR*(*F*^2^) = 0.176	H atoms treated by a mixture of independent and constrained refinement
*S* = 1.04	*w* = 1/[σ^2^(*F*_o_^2^) + (0.1*P*)^2^] where *P* = (*F*_o_^2^ + 2*F*_c_^2^)/3
2061 reflections	(Δ/σ)_max_ = 0.001
194 parameters	Δρ_max_ = 0.13 e Å^−^^3^
0 restraints	Δρ_min_ = −0.13 e Å^−^^3^

### Special details

**Table:**

Geometry. All e.s.d.'s (except the e.s.d. in the dihedral angle between two l.s. planes) are estimated using the full covariance matrix. The cell e.s.d.'s are taken into account individually in the estimation of e.s.d.'s in distances, angles and torsion angles; correlations between e.s.d.'s in cell parameters are only used when they are defined by crystal symmetry. An approximate (isotropic) treatment of cell e.s.d.'s is used for estimating e.s.d.'s involving l.s. planes.
Refinement. Refinement of *F*^2^ against ALL reflections. The weighted *R*-factor *wR* and goodness of fit *S* are based on *F*^2^, conventional *R*-factors *R* are based on *F*, with *F* set to zero for negative *F*^2^. The threshold expression of *F*^2^ > σ(*F*^2^) is used only for calculating *R*-factors(gt) *etc*. and is not relevant to the choice of reflections for refinement. *R*-factors based on *F*^2^ are statistically about twice as large as those based on *F*, and *R*- factors based on ALL data will be even larger.

### Fractional atomic coordinates and isotropic or equivalent isotropic displacement parameters (Å^2^)

**Table:**

	*x*	*y*	*z*	*U*_iso_*/*U*_eq_	
C1	0.2951 (5)	0.8186 (2)	0.07623 (17)	0.0481 (8)	
O1	−0.0143 (4)	0.8354 (2)	0.14362 (14)	0.0690 (8)	
N2	0.3009 (5)	0.7252 (2)	0.18825 (15)	0.0600 (8)	
C3	0.0114 (5)	0.9219 (3)	0.03213 (18)	0.0532 (8)	
H3A	−0.1196	0.9471	0.0389	0.064*	
C4	0.1183 (5)	0.9498 (2)	−0.03042 (18)	0.0528 (8)	
C2	0.0966 (5)	0.8582 (3)	0.08329 (17)	0.0508 (8)	
C11	0.3911 (6)	0.7562 (2)	0.13124 (19)	0.0569 (9)	
H11A	0.5264	0.7374	0.1249	0.068*	
C6	0.3994 (5)	0.8473 (2)	0.01367 (17)	0.0543 (8)	
H6A	0.5309	0.8225	0.0072	0.065*	
N1	0.0326 (4)	1.0119 (3)	−0.08228 (15)	0.0627 (8)	
C5	0.3174 (5)	0.9101 (3)	−0.03833 (17)	0.0535 (8)	
H5A	0.3928	0.9270	−0.0792	0.064*	
C12	0.4162 (5)	0.6759 (3)	0.24289 (18)	0.0555 (9)	
C9	0.1458 (6)	1.0453 (3)	−0.1467 (2)	0.0724 (11)	
H9A	0.0496	1.0655	−0.1839	0.087*	
H9B	0.2228	0.9858	−0.1648	0.087*	
N3	0.1521 (5)	0.5451 (3)	0.25389 (18)	0.0844 (11)	
H3B	0.1048	0.4891	0.2739	0.101*	
H3C	0.0871	0.5743	0.2190	0.101*	
C13	0.3329 (6)	0.5880 (3)	0.27784 (19)	0.0619 (10)	
C14	0.4406 (7)	0.5432 (3)	0.3335 (2)	0.0773 (12)	
H14A	0.3869	0.4849	0.3571	0.093*	
C16	0.7079 (7)	0.6677 (3)	0.3207 (2)	0.0798 (12)	
H16A	0.8329	0.6943	0.3352	0.096*	
C17	0.6027 (6)	0.7135 (3)	0.2645 (2)	0.0660 (10)	
H17A	0.6589	0.7710	0.2406	0.079*	
C7	−0.1687 (6)	1.0581 (3)	−0.0710 (2)	0.0727 (11)	
H7A	−0.2585	1.0041	−0.0522	0.087*	
H7B	−0.2223	1.0804	−0.1173	0.087*	
C10	0.2883 (6)	1.1352 (3)	−0.1340 (3)	0.0871 (13)	
H10A	0.3566	1.1524	−0.1782	0.131*	
H10B	0.3866	1.1155	−0.0982	0.131*	
H10C	0.2133	1.1952	−0.1175	0.131*	
C15	0.6240 (8)	0.5819 (4)	0.3550 (2)	0.0818 (13)	
H15A	0.6929	0.5502	0.3930	0.098*	
C8	−0.1708 (7)	1.1508 (4)	−0.0202 (3)	0.0933 (14)	
H8A	−0.3070	1.1767	−0.0154	0.140*	
H8B	−0.0854	1.2055	−0.0390	0.140*	
H8C	−0.1213	1.1291	0.0262	0.140*	
H1A	0.067 (7)	0.796 (4)	0.168 (2)	0.098 (16)*	

### Atomic displacement parameters (Å^2^)

**Table:**

	*U*^11^	*U*^22^	*U*^33^	*U*^12^	*U*^13^	*U*^23^
C1	0.0444 (16)	0.0430 (16)	0.0570 (19)	0.0037 (14)	0.0031 (16)	−0.0112 (15)
O1	0.0518 (14)	0.0809 (18)	0.0742 (17)	0.0140 (14)	0.0173 (13)	0.0094 (15)
N2	0.0612 (17)	0.0546 (16)	0.0643 (18)	0.0097 (15)	0.0110 (15)	0.0026 (15)
C3	0.0384 (16)	0.0548 (19)	0.067 (2)	0.0022 (15)	0.0021 (16)	−0.0071 (17)
C4	0.0463 (17)	0.0486 (17)	0.064 (2)	−0.0065 (15)	−0.0084 (16)	−0.0116 (16)
C2	0.0469 (17)	0.0475 (17)	0.0581 (19)	−0.0039 (16)	0.0080 (17)	−0.0092 (16)
C11	0.056 (2)	0.0458 (18)	0.069 (2)	0.0095 (16)	0.0007 (19)	−0.0099 (17)
C6	0.0449 (16)	0.0517 (19)	0.066 (2)	0.0060 (16)	0.0064 (17)	−0.0112 (16)
N1	0.0488 (16)	0.0772 (19)	0.0622 (17)	0.0011 (15)	−0.0072 (15)	0.0005 (16)
C5	0.0438 (17)	0.0570 (18)	0.060 (2)	−0.0009 (16)	0.0015 (17)	−0.0088 (17)
C12	0.0560 (19)	0.0485 (18)	0.062 (2)	0.0119 (17)	0.0056 (18)	−0.0012 (17)
C9	0.068 (2)	0.080 (3)	0.069 (2)	0.004 (2)	−0.014 (2)	0.004 (2)
N3	0.077 (2)	0.084 (2)	0.093 (2)	−0.0138 (19)	0.008 (2)	0.012 (2)
C13	0.062 (2)	0.056 (2)	0.067 (2)	0.0106 (19)	0.010 (2)	−0.0010 (18)
C14	0.084 (3)	0.067 (2)	0.080 (3)	0.019 (2)	0.020 (2)	0.012 (2)
C16	0.066 (2)	0.083 (3)	0.090 (3)	0.019 (2)	−0.004 (2)	0.000 (3)
C17	0.063 (2)	0.059 (2)	0.076 (2)	0.010 (2)	0.005 (2)	0.0051 (19)
C7	0.051 (2)	0.088 (3)	0.079 (2)	0.002 (2)	−0.017 (2)	0.008 (2)
C10	0.075 (3)	0.083 (3)	0.102 (3)	−0.011 (3)	−0.016 (3)	0.014 (2)
C15	0.091 (3)	0.078 (3)	0.076 (3)	0.029 (3)	0.000 (3)	0.017 (2)
C8	0.076 (3)	0.098 (3)	0.106 (3)	0.024 (3)	−0.001 (3)	−0.005 (3)

### Geometric parameters (Å, °)

**Table:**

C1—C6	1.397 (4)	C9—H9B	0.9700
C1—C2	1.407 (4)	N3—C13	1.383 (5)
C1—C11	1.438 (4)	N3—H3B	0.8600
O1—C2	1.367 (4)	N3—H3C	0.8600
O1—H1A	0.86 (5)	C13—C14	1.376 (5)
N2—C11	1.275 (4)	C14—C15	1.365 (6)
N2—C12	1.413 (4)	C14—H14A	0.9300
C3—C2	1.367 (4)	C16—C15	1.377 (6)
C3—C4	1.402 (5)	C16—C17	1.380 (5)
C3—H3A	0.9300	C16—H16A	0.9300
C4—N1	1.366 (4)	C17—H17A	0.9300
C4—C5	1.413 (5)	C7—C8	1.509 (5)
C11—H11A	0.9300	C7—H7A	0.9700
C6—C5	1.363 (4)	C7—H7B	0.9700
C6—H6A	0.9300	C10—H10A	0.9600
N1—C7	1.465 (5)	C10—H10B	0.9600
N1—C9	1.471 (4)	C10—H10C	0.9600
C5—H5A	0.9300	C15—H15A	0.9300
C12—C17	1.378 (5)	C8—H8A	0.9600
C12—C13	1.403 (5)	C8—H8B	0.9600
C9—C10	1.497 (5)	C8—H8C	0.9600
C9—H9A	0.9700		
			
C6—C1—C2	116.2 (3)	C13—N3—H3C	120.0
C6—C1—C11	121.1 (3)	H3B—N3—H3C	120.0
C2—C1—C11	122.7 (3)	C14—C13—N3	121.4 (4)
C2—O1—H1A	103 (3)	C14—C13—C12	118.3 (4)
C11—N2—C12	118.7 (3)	N3—C13—C12	120.2 (3)
C2—C3—C4	121.1 (3)	C15—C14—C13	121.8 (4)
C2—C3—H3A	119.4	C15—C14—H14A	119.1
C4—C3—H3A	119.4	C13—C14—H14A	119.1
N1—C4—C3	121.4 (3)	C15—C16—C17	118.7 (4)
N1—C4—C5	121.1 (3)	C15—C16—H16A	120.6
C3—C4—C5	117.5 (3)	C17—C16—H16A	120.6
O1—C2—C3	118.2 (3)	C12—C17—C16	121.4 (4)
O1—C2—C1	119.8 (3)	C12—C17—H17A	119.3
C3—C2—C1	121.9 (3)	C16—C17—H17A	119.3
N2—C11—C1	123.5 (3)	N1—C7—C8	114.2 (3)
N2—C11—H11A	118.2	N1—C7—H7A	108.7
C1—C11—H11A	118.2	C8—C7—H7A	108.7
C5—C6—C1	123.0 (3)	N1—C7—H7B	108.7
C5—C6—H6A	118.5	C8—C7—H7B	108.7
C1—C6—H6A	118.5	H7A—C7—H7B	107.6
C4—N1—C7	120.3 (3)	C9—C10—H10A	109.5
C4—N1—C9	121.9 (3)	C9—C10—H10B	109.5
C7—N1—C9	117.4 (3)	H10A—C10—H10B	109.5
C6—C5—C4	120.2 (3)	C9—C10—H10C	109.5
C6—C5—H5A	119.9	H10A—C10—H10C	109.5
C4—C5—H5A	119.9	H10B—C10—H10C	109.5
C17—C12—C13	119.4 (3)	C14—C15—C16	120.4 (4)
C17—C12—N2	122.3 (3)	C14—C15—H15A	119.8
C13—C12—N2	118.3 (3)	C16—C15—H15A	119.8
N1—C9—C10	114.2 (3)	C7—C8—H8A	109.5
N1—C9—H9A	108.7	C7—C8—H8B	109.5
C10—C9—H9A	108.7	H8A—C8—H8B	109.5
N1—C9—H9B	108.7	C7—C8—H8C	109.5
C10—C9—H9B	108.7	H8A—C8—H8C	109.5
H9A—C9—H9B	107.6	H8B—C8—H8C	109.5
C13—N3—H3B	120.0		

### Hydrogen-bond geometry (Å, °)

**Table:**

*D*—H···*A*	*D*—H	H···*A*	*D*···*A*	*D*—H···*A*
N3—H3B···O1^i^	0.86	2.55	3.395 (4)	167
O1—H1A···N2	0.86 (5)	1.82 (5)	2.638 (4)	157 (4)

Symmetry codes: (i) −*x*, *y*−1/2, −*z*+1/2.

## References

[bb1] Chen, Z. H., Morimoto, H., Matsunaga, S. & Shibasaki, M. (2008). *J. Am. Chem. Soc. ***130**, 2170–2171. 10.1021/ja710398q 18225906

[bb2] Elmah, A., Kabak, M. & Elerman, Y. (1999). *J. Mol. Struct. ***484**, 229–234.

[bb3] May, J. P., Ting, R., Lermer, L., Thomas, J. M., Roupioz, Y. & Perrin, D. M. (2004). *J. Am. Chem. Soc. ***126**, 4145–4156. 10.1021/ja037625s 15053604

[bb4] Rigaku (2005). *CrystalClear* Rigaku Corporation, Tokyo, Japan.

[bb5] Sheldrick, G. M. (2008). *Acta Cryst.* A**64**, 112–122. 10.1107/S0108767307043930 18156677

[bb6] Weber, B., Tandon, R. & Himsl, D. (2007). *Z. Anorg. Allg. Chem.* **633**, 1159–1162.

